# Strengthening Routine Health Information Systems to Target Malaria Control Implementation and Optimize Evaluation of Impact

**DOI:** 10.4269/ajtmh.17-0581

**Published:** 2017-09-27

**Authors:** Scott Filler

**Affiliations:** Senior Disease Coordinator, The Global Fund to Fight AIDS, Tuberculosis and Malaria, Vernier, Geneva, Switzerland

The Global Fund provides 50% of all international financing for malaria and since 2000 has invested more than US$8 billion in programs that prevent and treat the disease. The Global Fund supports a comprehensive approach that combines education, prevention, diagnosis, and treatment. The work of the Global Fund is based on four principles—partnership, country-ownership, performance based financing, and transparency—empowering implementers to lead the response to human immunodeficiency virus, tuberculosis, and malaria, supported by a diverse range of partners in the health sector. The Global Fund plays a critically important role, and it is imperative that funding is invested for maximum impact, supporting the implementation of programs in the most effective way possible.

Good data are essential for resource allocation, programmatic planning, and evaluation of program impact. Systematic efforts and long-term investments in routine data systems are needed to improve the availability and quality of data for analysis, dissemination, and use in strategic decision-making; and to provide capacity for better targeting of programs, improving quality, and providing for more efficient service delivery. Acknowledging this, the Global Fund has committed to be a part of the Health Data Collaborative and will continue to maximize existing efforts and resources from all global and domestic partners to improve data availability, data quality, and data use at the national, local and community level through coordinated investments in national data systems. More specifically, the Global Fund will systematically invest in country-specific surveillance, monitoring, and evaluation plans to inform program design, track program implementation, and measure impact. These investments should help to ensure that countries have systems in place to generate the comprehensive data needed to target and manage their health programs. In addition, Global Fund investment in country data systems and tools for assessing data quality will allow for better policy and decision making to maximize program efficiency and quality. This targeted effort will also include enabling communities and local providers to access, use, and act on these data to highlight issues with program quality and barriers to accessing services.

The impact of Global Fund-supported programs is further strengthened by planning interventions together with governments, partners, and critical bilateral programs. One concrete example of this collaboration is program evaluation: systematically collecting, analyzing, and using information to answer questions regarding malaria programs. Evaluation of program effectiveness and efficiency outside national reviews may be necessary, and all partner-led evaluations (e.g., President’s Malaria Initiative impact evaluations) should be taken into consideration when allocating scarce resources.

The Global Fund commends the work of this supplement’s authors because these articles demonstrate the collective commitment to support a broad range of data collection efforts. Nationwide surveys are extremely helpful to monitor changes in coverage of key malaria control interventions (such as insecticide-treated net ownership and use) and to help measure their impact, particularly on all-cause child mortality, malaria parasitemia, and anemia. However, more emphasis must be placed on improving completeness, timeliness, representativeness, and accuracy of routine health information systems data for evaluating causal effects of health programs.

